# MicroRNA and metabolomics signatures for adrenomyeloneuropathy disease severity

**DOI:** 10.1002/jmd2.12323

**Published:** 2022-08-22

**Authors:** Bela Rui Turk, Laila Marie Poisson, Christina Linnea Nemeth, Jordan Goodman, Ann B. Moser, Richard Owen Jones, Ali Fatemi, Jaspreet Singh

**Affiliations:** ^1^ Moser Center for Leukodystrophies, Kennedy Krieger Institute Johns Hopkins Medical Institutions Baltimore Maryland USA; ^2^ Department of Public Health Sciences Henry Ford Health System Detroit Michigan USA; ^3^ Department of Neurology Henry Ford Health System Detroit Michigan USA

**Keywords:** adrenoleukodystrophy, adrenomyeloneuropathy, biomarker, leukodystrophy, metabolomics, micro‐RNA

## Abstract

Adrenomyeloneuropathy (AMN), the slow progressive phenotype of adrenoleukodystrophy (ALD), has no clinical plasma biomarker for disease progression. This feasibility study aimed to determine whether metabolomics and micro‐RNA in blood plasma provide a potential source of biomarkers for AMN disease severity. Metabolomics and RNA‐seq were performed on AMN and healthy human blood plasma. Biomarker discovery and pathway analyses were performed using clustering, Kyoto Encyclopedia of Genes and Genomes (KEGG) pathway analysis, and regression against patient's clinical Expanded Disability Status Score (EDSS). Fourteen AMN and six healthy control samples were analyzed. AMN showed strong disease‐severity‐specific metabolic and miRNA clustering signatures. Strong, significant clinical correlations were shown for 7‐alpha‐hydroxy‐3‐oxo‐4‐cholestenoate (7‐HOCA) (*r* ^2^ = 0.83, *p* < 0.00001), dehydroepiandrosterone sulfate (DHEA‐S; *r* ^2^ = 0.82, *p* < 0.00001), hypoxanthine (*r* ^2^ = 0.82, *p* < 0.00001), as well as miRNA‐432‐5p (*r* ^2^ = 0.68, *p* < 0.00001). KEGG pathway comparison of mild versus severe disease identified affected downstream systems: GAREM, IGF‐1, CALCRL, SMAD2&3, glutathione peroxidase, LDH, and NOS. This feasibility study demonstrates that miRNA and metabolomics are a source of potential plasma biomarkers for disease severity in AMN, providing both a disease signature and individual markers with strong clinical correlations. Network analyses of affected systems implicate differentially altered vascular, inflammatory, and oxidative stress pathways, suggesting disease‐severity‐specific mechanisms as a function of disease severity.

**Synopsis**

Micro‐RNA and metabolomics markers in adrenomyeloneuropathy blood plasma provide a rich potential source of biomarkers, correlating cross‐sectionally with disease progression.

## 1 INTRODUCTION

X‐linked adrenoleukodystrophy (ALD; MIM #300100) is an inborn error of metabolism, due to defects in the peroxisomal membrane transporter protein. Close to 900 pathogenic variants of the underlying *ABCD1* gene have been identified (https://adrenoleukodystrophy.info), which lead to peroxisomal reduced fatty acid beta‐oxidation and hallmark impaired degradation and accumulation of very long chain fatty acids (VLCFA) in all tissues.^1^ ALD presents in males as either a rapid progressive fatal cerebral demyelination (cALD) in young boys or adulthood or/and a slowly progressive spinal cord myelopathy, adrenomyeloneuropathy (AMN), starting in the 20s. AMN presents with a spectrum of slow, and variably progressive symptoms. Both central and peripheral nervous system pathology lead to motor and sensory dysfunction: spasticity, stiffness, weakness, sensory ataxia and gait impairment, as well as sexual and bladder dysfunction. Furthermore, peripheral neuropathy presenting with paresthesia, pain, and adrenal insufficiency is common.^1^ Investigation of VLCFA and *ABCD1* pathogenic variant can confirm X‐ALD diagnosis in a suspected male patient; however, it cannot predict the clinical course of the disease progression (AMN or cALD). In the absence of a direct biomarker or genetic correlate, many groups have looked toward environmental factors, modifier genes, transcriptomics, lipidomics, and microRNA (miRNA) profiling to understand the heterogeneity of ALD and AMN.^1^, ^2^, ^3^, ^4^ As biomarker discovery is a principal endeavor for AMN, we measured candidate biomarkers against the standard neurological severity variable, namely the Expanded Disability Status Scale (EDSS), originally designed to quantify disability in multiple sclerosis.^5^

Metabolomics and miRNA expression allows for the quantification of the entire spectrum of potential markers in biofluids, which captures the functional state of the organism at a given time point. Omics‐based markers may serve two functions: as a wide net for biomarker discovery, and to stratify AMN phenotypes through biochemical signatures, which may help cluster patients yielding insight into underlying mechanisms. MiRNAs are small, single‐stranded noncoding RNAs that play a role in regulating gene expression, or in post‐translational epigenetic mechanisms.^4^ Specific miRNA have been implicated in neurological disease processes, and may, in addition to ‐omics‐based approaches, provide discovery insight into potential disease mechanisms.

In this feasibility study, we sought to determine whether metabolomics and miRNA‐seq‐based approaches could be used to characterize, cluster, and identify potential plasma biomarkers for disease severity in AMN.

## 2 METHODS

### 2.1 Patient recruitment and inclusion criteria

All patients and controls were seen at the Kennedy Krieger Institute and had a confirmed biochemical diagnosis of ALD. Patient data and samples were collected during routine physician visits between 2015 and 2018 and stored at −80°C until use. Male patients over the age of 18, with a genetically confirmed diagnosis of ALD, and pure AMN phenotype were included. At the time of sampling, patients had AMN without cerebral involvement. Patients with a pure AMN phenotype without cerebral involvement have an EDSS of at least 1 and do not have evidence of cerebral demyelination on brain MRI at the time of assessment. AMN patients were categorized as mild (EDSS 1–3) or severe (EDSS 4–9). Controls were non‐ALD healthy controls with no functional neurological deficit.

### 2.2 Metabolomics

Global metabolomics profiling by ultraperformance liquid chromatography mass spectrometry (UPLC‐MS; Metabolon Inc.) was performed on blood plasma samples (200 μl each) from healthy controls and AMN subjects. The per‐metabolite data were median‐scaled and minimum‐value imputed. Data were log2‐transformed, for interpretation as fold change relative to the median. Analysis of variance (ANOVA) per metabolite was used to screen for differences in mean intensity across diagnosis groups. *p* values from the global F‐test were converted to false discovery rate (FDR, Benjamini–Hochberg) *q*‐values and the threshold for selection set at FDR ≤0.05. Post hoc testing of two‐group comparisons was conducted for the selected metabolites to determine the differences (*p* ≤ 0.05).

### 2.3 miRNA

Plasma samples (200 μl) were processed for next‐generation sequencing at the USC Norris Cancer Center Molecular Genomics Core. Samples were extracted using QIAGEN's miRNeasy Kit following the manufacturer's protocol. Libraries were prepared from extracted total RNA enriched in miRNA using QIAseq miRNA Library Kit (QIAGEN). The libraries were sequenced on the Illumina Nextseq500 platform on 1x75 read length. Raw miRNA‐sequencing reads in FASTQ files were uploaded to the QIAGEN GeneGlobe Data Analysis Center for primary quantification. The 3′ adapter of sequencing reads and low‐quality bases were first trimmed using Cutadapt (https://cutadapt.readthedocs.io/en/stable/). Reads with less than 16 bp insert sequences or less than 10 bp unique molecular index (UMI) sequences were also removed. The insert sequence reads were then aligned to human GRCh38 reference databases (mapping to miRbase mature, miRbase hairpin, piRNA, rRNA, tRNA, mRNA, and other RNA based on miRBase V21, piRNABank, and Genome Reference Consortium GRCh38) using Bowtie (http://bowtie-bio.sourceforge.net/index.shtml). Read and UMI counts for each RNA type were subsequently quantified from the mapping results. Assessment of differential expression used negative binomial modeling (DESeq2 package) for each of the two‐sample comparisons of the diagnosis groups, with FDR controlled at 5% (Benjamini–Hochberg). Normalized counts were exported for use in graphing.

### 2.4 Cluster analyses

Hierarchical clustering using Pearson's correlation and complete linkage was used to order the substrates. Partial least‐squares discriminant analysis (PLS‐DA) was performed on metabolomics and miRNA separately.

### 2.5 Pathway analysis

*Metabolites*: Enrichment of changes in 80 metabolic pathways from the Kyoto Encyclopedia of Genes and Genomes (KEGG, for homo sapiens) was assessed by Fisher's exact test. An additional measure of the level of impact the alterations have on a pathway was estimated using betweenness‐centrality and was performed in Metaboanalyst 4.0 (July 2019). The list of measured metabolites was imported into Ingenuity Pathway Analysis (IPA), mapped by HMDB number, and core analysis was performed on the differential lists of metabolites.

*miRNA*: Enrichment of biofunctions and disease pathways was performed using IPA core analysis. The full set of miRNA was used as the reference. Terms meeting the threshold of FDR < 0.05 were retained.

### 2.6 Clinical score regression analyses

A biomarker discovery pipeline was designed: First, ordinary least‐squares regression and mixed‐linear modeling were conducted on all metabolites and miRNA against the individual EDSS score of AMN only (healthy controls excluded from regression), and biomarkers were ranked by coefficient of determination (*r* ^2^). Second, biomarkers with any overlap with severe AMN, or over 20% overlap range between control and any AMN were excluded. Xenobiotics and metabolites with known exogenous or dietary sources were excluded. Significance testing was performed using omnibus F‐testing within analyses of variance. Adjusted *R* ^2^ values are reported. Bonferroni adjusted *p* values for multiple comparisons are used to determine levels of significance. Regression analyses were performed in python (3.8.0), with the packages pandas (1.0.1), seaborn (0.10.0), statsmodels (0.11.0), matplotlib (3.1.3), scipy (1.4.1), and numpy (1.18.1).

### 2.7 Age analyses

Ordinary least squares and multiple regression analyses were performed to determine whether biomarker performance may be attributed to patient age, within AMN. Controls were regressed separately. Additionally, EDSS was regressed against age.

## 3 RESULTS

### 3.1 Patient characteristics

Fourteen AMN and six healthy control subjects were included in the study. The mean age for AMN subjects was 46.4 years (range: 30–70; SD: 13.23) and 42.2 years for controls (range: 24–73; SD: 18.81). AMN patients were stratified by disease severity into mild (EDSS ≤3, *n* = 6) and severe (EDSS 4+, *n* = 8). Mean EDSS of the mild group was 2 (1–3) and severe group was 5.5 (4–7). All AMN patients had a diagnosis of adrenal insufficiency. Demographic data are shown in Table S1.

### 3.2 Molecular signatures in categoric analyses by disease severity

**FIGURE 1:**
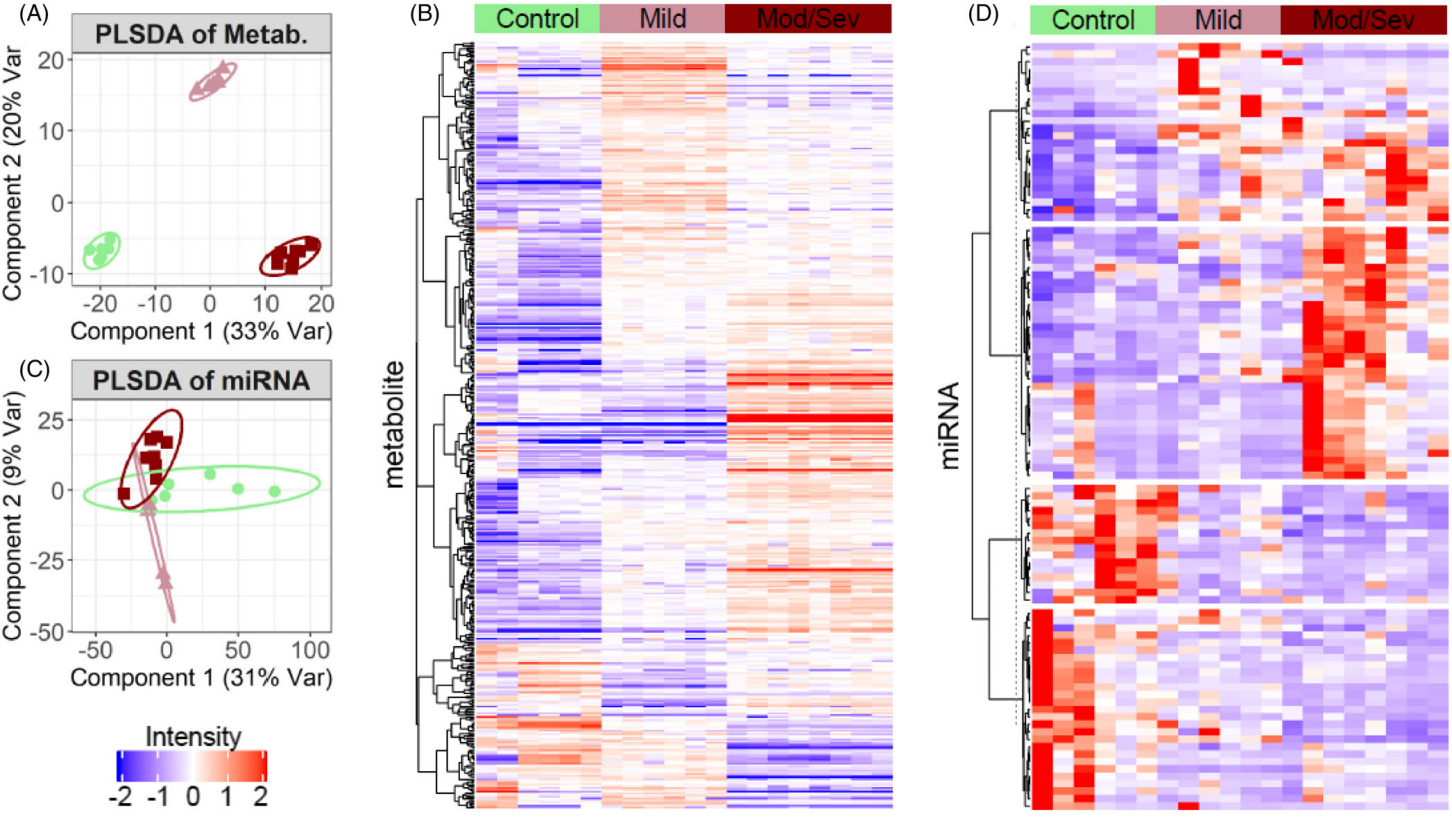
Partial least‐squares discriminant analysis (PLSDA) shows metabolite (A) and micro‐RNA (C) clustering. Clustered heatmaps of differential (B) metabolomics and (D) micro‐RNA between control, mild, and moderate/severe adrenomyeloneuropathy (AMN)

Untargeted metabolomics were performed on plasma samples using UPLC‐MS. A total of 826 metabolites were identified and quantified by LC/MS. Of these, 170 were identified as Xenobiotics and excluded. When examined by partial least squares discriminant analysis (PLS‐DA), metabolic phenotypes of plasma from control and AMN patients demonstrate that strong clustering by disease severity group can be achieved (Figure 1A). Analysis of differential mean intensity between controls, mild AMN, and moderate/severe AMN plasma samples identified 402 metabolites (ANOVA *F*‐test, FDR < 0.05). Most identified metabolites were increased in the AMN groups, though the patterns were not exclusively monotonic with severity (Figure 1B).

**FIGURE 2:**
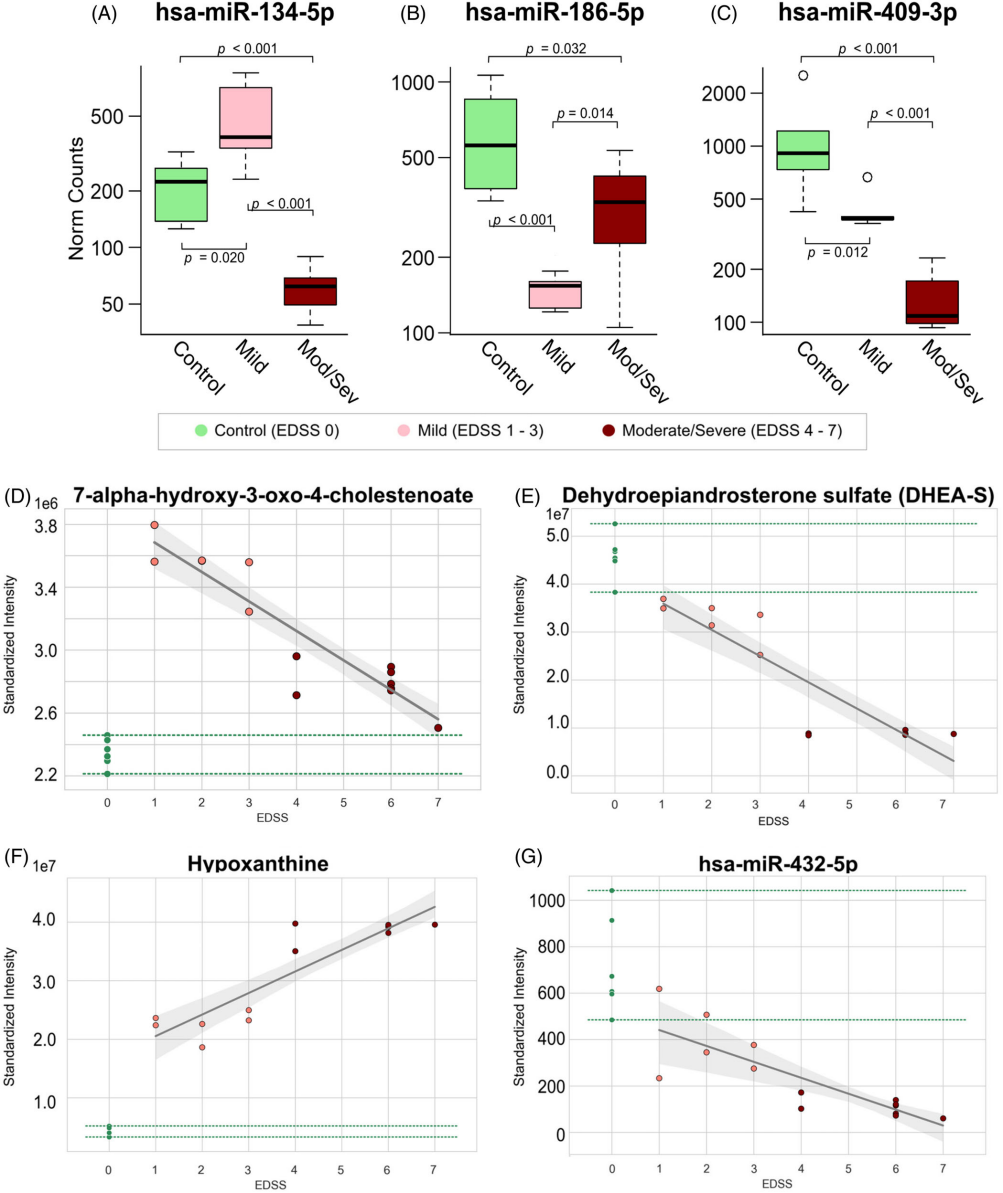
Micro‐RNAs (A) 134‐5p, (B) 186‐5p, and (C) 409‐3p significantly differentiate control, mild, and moderate/severe AMN. Linear regression analyses of molecular intensity within AMN against the Kurtzke Expanded Disability Status Scale (EDSS; range 1–7 included). Scatterplots are shown for molecules with significant linear association and minimal overlap with control (EDSS 0): (D) 7‐alpha‐hydroxy‐3‐oxo‐4‐cholestenoate (7‐HOCA), (E) dehydroepiandrosterone sulfate (DHEA‐S), (F) hypoxanthine, and (G) micro‐RNA 432‐5p. The estimated linear relationship is plotted with a solid black and the 95% confidence bands on this line are shown in gray. The range of observed molecular intensity for controls is denoted with green‐dashed lines

Profiling for miRNA in control and AMN plasma was performed using high‐throughput RNA‐seq, with 2545 miRNAs identified for analysis in the plasma samples. PLS‐DA analysis of miRNA revealed a good separation between control and AMN plasma samples (Figure 1C). Two‐group comparisons of miRNA counts between controls, mild AMN, and severe AMN plasma samples identified 101 differential miRNAs (negative binomial modeling, FDR < 0.05). Clustered metabolites and miRNA expression are shown for each group in Figure 1B,D. The miRNAs miR‐134‐5p, miR‐186‐5p, and miR‐409‐3p are significantly different between all three groups: control, mild, and moderate/severe AMN (FDR < 0.05; Figure 2A–C). MiR‐409‐3p shows lower AMN expression creating a monotonic trend (Figure 2C).

### 3.3 Identifying potential biomarkers by EDSS

In a first step, linear regression between molecular intensity and EDSS score (range: 1–7) in AMN was performed. Then, to identify potential biomarkers of disease severity which only show abnormal levels in AMN, those molecules that had intensity (expression) level overlap of more than 20% between control and AMN cases were excluded. Exogenous‐ and dietary metabolites were also excluded. There were three metabolites and one miRNA retained with strong clinical correlations (Figure 2D–G): 7‐alpha‐hydroxy‐3‐oxo‐4‐cholestenoate (7‐HOCA) (*r* ^2^ = 0.83, *p* = 0.0002), dehydroepiandrosterone sulfate (DHEA‐S) (*r* ^2^ = 0.82, *p* = 0.0002), hypoxanthine (*r* ^2^ = 0.82, *p* = 0.0002), and miRNA: 432‐5p (*r* ^2^ = 0.68, *p* = 0.007). A selected set of metabolites and miRNAs identified in the primary categoric ANOVA group analysis (control vs. mild vs. moderate/severe AMN) were regressed against EDSS and are shown in Figure S1.

These include asparagine (*r* ^2^ = 0.63, *p* = 0.002), aspartate (*r* ^2^ = 0.78, *p* = 0.0002), cystein‐glutathione disulfide (*r* ^2^ = 0.79, *p* = 0.0002), glutamate (*r* ^2^ = 0.80, *p* = 0.0002), glutamine (*r* ^2^ = 0.02, *p* = 0.63), glycine (*r* ^2^ = 0.49, *p* = 0.01), sarcosine (*r* ^2^ = 0.73, *p* = 0.0005), threonine (*r* ^2^ = 0.30, 0.06), miR‐134‐5p (*r* ^2^ = 0.52, *p* = 0.69), miR‐186‐5p (*r* ^2^ = 0.44, *p* = 0.98), miR‐409‐3p (*r* ^2^ = 0.66, *p* = 0.28), and miR‐451‐a (*r* ^2^ = 0.57, *p* = 0.7).

### 3.4 Biomarkers as a function of age

To assess if age is confounding the molecular relationships with EDSS, we modeled the linear relationship between age and EDSS (range: 1–7) within AMN. There was a trend to a positive correlation (*r* ^2^ = 0.25, *p* = 0.06; Figure S2). No significant correlations between age and metabolite were found in AMN or controls.

### 3.5 Follow‐up analyses for phenotype‐switch to cerebral disease

**FIGURE 3:**
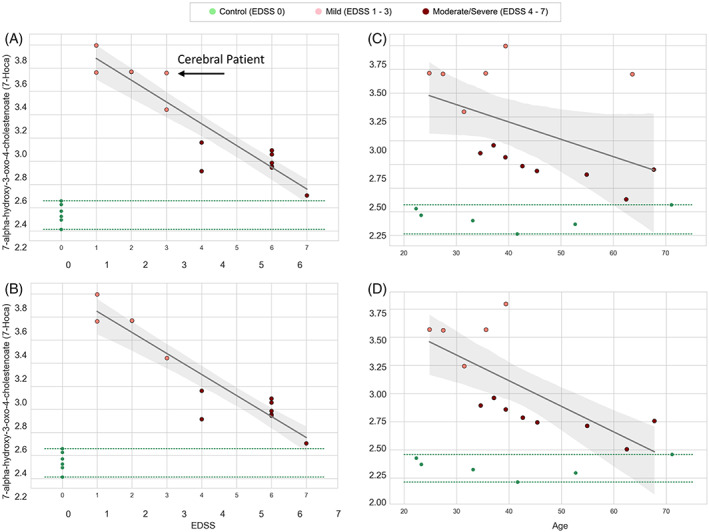
Sensitivity analyses regression modeling within AMN for EDSS versus 7‐HOCA (A) with all patients, (B) excluding potential outlier. (C) Age versus 7‐HOCA with all patients and (D) excluding outlier. Arrow indicates potential phenotypic outlier diagnosed with cerebral disease 4 years after the sample date

Clinical records for AMN patients were obtained from the sample date to Jan 2021. One mild AMN patient with EDSS 3 was sampled at age 63 in 2014. Only this patient saw diagnosis of cerebral ALD in 2019. All regression analyses were repeated excluding the cerebral patient in order to assess the sensitivity of the analysis to this pre‐progression patient sample. The top metabolites identified by the regression models with and without this case remained the same (Figure 2D–G). Excluding the patient from regression analyses does not alter the significant correlation between 7‐HOCA and EDSS versus excluded patient 7‐HOCA and EDSS (*r* ^2^ = 0.83, *p* < 0.00001; Figure 3A,B). However, after excluding the patient that progressed to cALD, we see that 7‐HOCA was identified as strongly correlated with age (*r* ^2^ = 0.50, *p* = 0.007), which was not the case when the patient was included in the model (*r* ^2^ = 0.25, *p* = 0.08), shown in (Figure 3C,D). The patient's 7‐HOCA value is similar to those in young AMN.

### 3.6 Pathway analysis as a function of disease severity

KEGG enrichment was assessed for metabolites that differed between mild and moderate/severe AMN (ANOVA FDR < 0.05, follow‐up two‐group test *p* < 0.05), in order to determine which pathways are affected as a function of disease severity. KEGG analysis identified the following systems significantly altered by highest impact, that is the changed metabolites sit in key junction points of the pathway (Figure S3A): (1) taurine and hypotaurine; (2) d‐glutamine and d‐glutamate; (3) phenylalanine, tyrosine, and tryptophane biosynthesis; (4) vitamin B6; (5) alanine, aspartate, and glutamate; (6) glycine, serine, and threonine; and (7) beta‐alanine. To examine biological functions related to altered miRNA between mild and moderate/severe AMN, pathway enrichment was conducted using the ingenuity knowledgebase. Inflammatory disease, inflammatory response, and neurological disease toped the ranked list of affected functions (Figure S3B).

**FIGURE 4:**
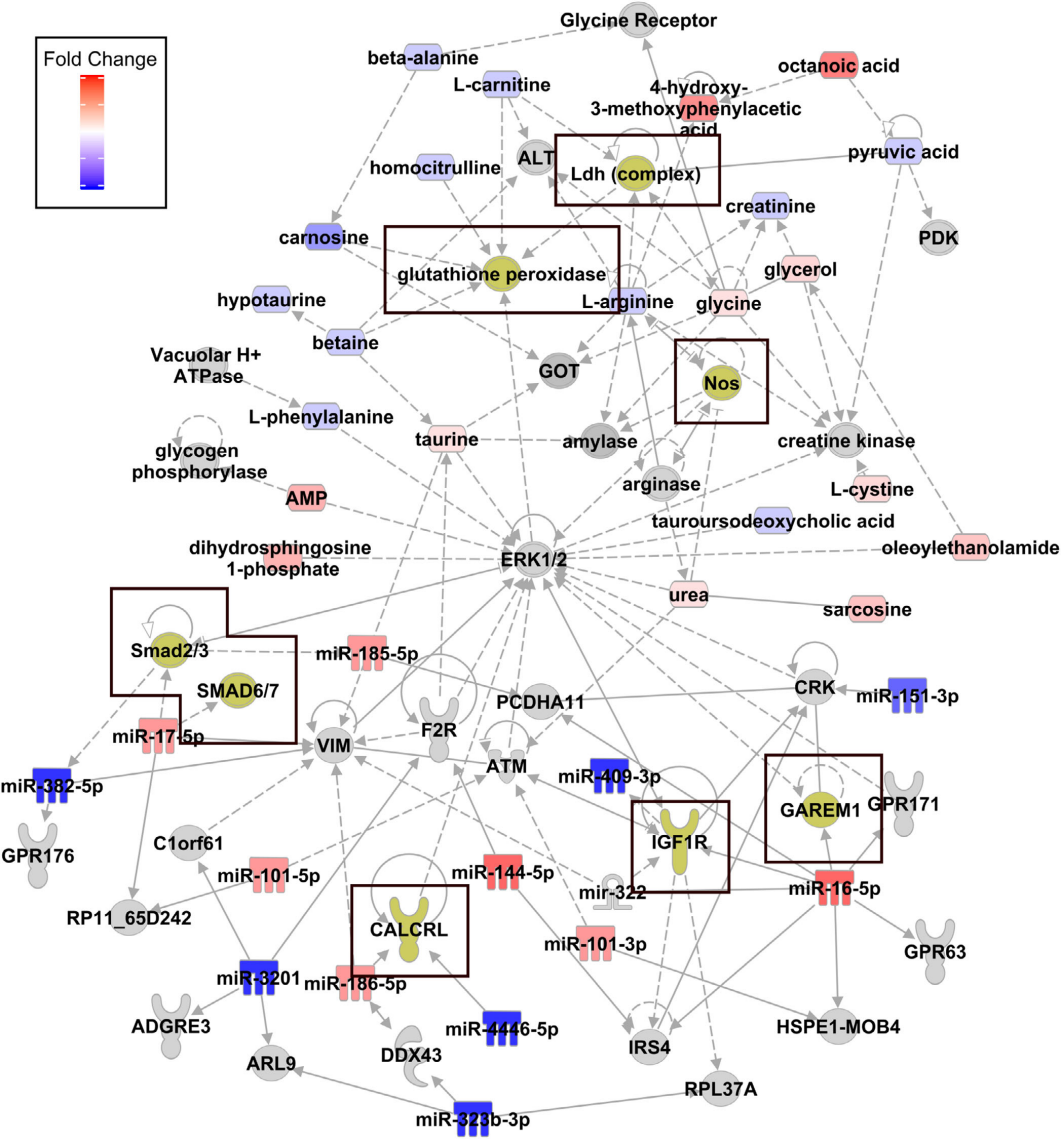
Combined micro‐RNA and metabolite network analyses identify downstream systems and targets (yellow). Upregulated (red) and downregulated (blue) substrates are linked directly (solid gray arrow), or indirectly (dotted gray arrow)

We then conducted integrated network analyses to generate a pathway map that combined both significantly changed miRNAs and metabolites between mild and moderate/severe AMN. Central to altered miRNA were signaling pathways SMAD 2/3, SMAD 6/7, CALCRL, IGF1‐R, and GAREM1. Central to the altered metabolites were inflammatory pathways NOS, LDH complex, and glutathione peroxide. ERK1/2 was identified as a pathway node joining the miRNA and metabolites networks (Figure 4).

## 4 DISCUSSION

Recent advances in drug discovery have resulted in identification of several potential therapeutics for adult men with AMN; however, clinical trials have been hampered by the rarity of the disease, resulting in limited availability of natural history data and a highly variable rate of progression in AMN, with some subjects progressive over years and others remaining stable for decades. There is a great need to identify markers that correlate with disease severity in AMN, which could be used for stratification of patients for clinical trials but also may serve as predictors of outcome in future prospective studies.

In this feasibility study, UPLC–MS‐based metabolomics and RNAseq‐based quantification of miRNA expression performed in blood plasma of a cohort of well phenotyped AMN patients demonstrate that these blood parameters have the potential to serve as biomarkers reflecting disease severity. Encouragingly, both metabolites and miRNA showed strong clustering within groups, as confirmed by PLS‐DA. This first step identified potential metabolic and miRNA signatures, not just separating AMN from healthy controls but differentiating mild from severe AMN, as shown in Figure 2.

7‐HOCA is the main metabolite of oxysterol 27‐hydroxycholesterol (27‐OHC) in the brain.^6^ An increase in 7‐HOCA has been shown to reflect damage to the blood–brain barrier when measured in cerebrospinal fluid.^7^ In our data, plasma 7‐HOCA is high in mild, and low in severe in AMN (*r* ^2^ = 0.83, *p* < 0.0001). However, regressed against age, 7‐HOCA first showed a trend toward a weak inverse correlation (*r* ^2^ = 0.25, *p* = 0.08). Upon closer examination of these data, we identified an outlier patient greatly reducing the strength of the correlation. The mild AMN outlier (EDSS 3, age 63) demonstrated a much higher 7‐HOCA value than his later‐age counterparts, similar to those of young AMN patients. Interestingly, the outlier patient was the only patient in this cohort found to have developed cerebral disease, diagnosed in 2019, 4 years after the sample date in 2015. Repeat sensitivity analyses excluding the outlier showed a significant correlation with age (*r* ^2^ = 0.50, *p* = 0.04) within AMN, without affecting the significant correlation to EDSS. In controls, there was no significant correlation between 7‐HOCA and age. Further studies are needed to determine whether 7‐HOCA has any predictive value in identifying individuals who are at risk of developing cALD later in life.

DHEA‐S is an endogenous androstane steroid and regulator of peroxisomal function, shown to induce proliferation and stimulate peroxisomal enzyme activity involved in fatty acid metabolism.^8^, ^9^, ^10^ AMN patients commonly present with primary adrenocortical insufficiency, showing low levels of cortisol, DHEA and DHEA‐S. Decreased testosterone levels have also been reported in ALD.^11^ We show low DHEA‐S in severe AMN, and no correlation with age (*r* ^2^ = 0.11, *p* = 0.16). However, DHEA‐S is transiently high in blood plasma following DHEA administration, and our patients may be on DHEA supplementation, which may be a confounder.^12^

Hypoxanthine is oxidized by xanthine oxidase to uric acid, a process which generates radical oxygen species (ROS).^13^, ^14^ Hypoxanthine has been shown to induce endothelial dysfunction via ROS production and induce apoptosis preventable by N‐acetylcysteine pretreatment.^14^ We have previously shown that antioxidant function of peripheral blood cells shows a phenotype‐specific response between different ALD phenotypes.^15^, ^16^ While plasma hypoxanthine is also shown to increase with physical activity which we have not measured in our patients,^17^ our sample data shows higher levels of hypoxanthine in patients with an EDSS of 6+. Considering the severe gait impairment of these patients, we cautiously assume that the physical activity level of these patients may be lower than those with mild disease. In summary, we believe that there is strong rationale for the abnormal elevation of hypoxanthine as a function of disease severity, supporting the potential of this metabolite as a plasma biomarker in AMN.

We have previously shown alterations in glutathione peroxidase metabolism, previous attempts to discriminate between ALD phenotypes.^15^ While our metabolomics panel does not capture total glutathione, we report increased cysteine‐glutathione disulfide, the oxidized form, in severe versus mild AMN.

Two previous studies, including one from our group, have reported miRNA changes in patient cells.^4^, ^18^ However, no plasma miRNA has been reported for AMN or ALD phenotypes. Our categorical analysis identified three miRNAs driving the signatures differentiating controls and AMN severity: miR‐134‐5p, miR‐186‐5p, and miR‐409‐3p, as well as miR‐432‐5p.

MiR‐134‐5p is shown to contribute to synapto‐dendritic plasticity.^19^ MiR‐134‐5p decrease is reported in a neuropathic pain rat model of chronic sciatic nerve injury.^20^ Inducing miR‐134‐5p overexpression alleviated neuropathic pain and decreased the expression of inflammatory cytokines.^20^, ^21^, ^22^ Future analysis will be needed to compare this miRNA with clinical outcome measures such as pain scales in AMN subjects to determine whether the MiR‐134‐5p reduction seen in severe AMN is associated with severe peripheral neuropathy.

MiR‐409‐3p is a reported biomarker in CSF for Parkinson's disease,^23^ and in plasma differentiating the phenotypes of Rett syndrome.^24^ Overexpression is shown to downregulate inflammatory cytokine production by astrocytes in the autoimmune encephalitis mouse model by reducing SOCS3 expression,^25^ a protein also found elevated in AMN patient PBMCs.^26^ We show low miR‐409‐3p in severe AMN.

High miR‐186‐5p is associated with improved neurological outcomes in the spinal cord ischemia–reperfusion injury model.^27^ We show decreased miR‐186‐5p in AMN versus control, with lower expression in the mild versus the moderate/severe group.

We show decreased miR‐432‐5p in severe AMN. A reported downstream target is CXC Chemokine ligand 5 (CXCL5),^28^ a leukocyte chemokine and potential inducer of microglial activation, increased BBB damage, and white matter injury.^29^

Combined IPA and KEGG network comparative analyses between mild and moderate/severe disease patients identified downstream pathways, previously implicated in ALD pathology literature: IGF‐1 dysfunction has been observed in both neonatal and early childhood (noncerebral) ALD patient fibroblasts^30^ and in reduced insulin signaling in the ABCD1‐KO mouse spinal cord.^31^ SMADs 2/3 is downstream of the TGFβ family, which is increased in ABCD1‐deficient vascular endothelia.^32^ We also show reduced miR‐17‐5p, the inhibitory mediator of SMAD 2/3 in severe AMN. GAREM is a ubiquitously expressed signal in the endothelial growth factor (EGF) pathway and contributes to neurite outgrowth in neuroblastoma cells.^33^ We also show reduced MiR‐16‐5p in severe AMN, an upstream effector of GAREM.

While network analyses comparing disease severity profiles implicate interesting vascular, inflammatory, and oxidative stress pathways, these differences must be explored in cellular models for mechanistic studies in the future. One limitation of the study are exogenous effects on the human metabolome. Dietary effects were not accounted for in these patients and should be considered in future studies, as well as menstrual cycle in female heterozygote AMN.

In summary, our data adds to previous reports showing differences between AMN patients and healthy controls,^26^ and demonstrates the feasibility of using metabolomics and miRNA to identify potential biomarkers of clinical disease severity in AMN. This first step in identifying potential biomarkers should be followed by longitudinal validation studies in larger cohorts. Future ‐omics and RNA‐seq‐based studies should also include asymptomatic AMN patients as well as asymptomatic and symptomatic heterozygote female AMN patients.

## AUTHOR CONTRIBUTIONS

Jaspreet Singh conceived the idea, and provided direction and funding for the project. Bela Rui Turk and Laila Marie Poisson designed and performed analyses and drafted the manuscript and shared the role of first authorship. Christina Linnea Nemeth collected and processed tissue samples and reviewed the manuscript. Jordan Goodman and Richard Owen Jones collected patient data and reviewed the manuscript. Ann B. Moser collected and processed tissue samples and reviewed the manuscript. Ali Fatemi and Jaspreet Singh designed the study and reviewed the manuscript. Jaspreet Singh serves as a guarantor for the article, accepts full responsibility for the work and the conduct of the study, had access to the data, and controlled the decision to publish.

## FUNDING INFORMATION

This study was supported by R21 NS104560 from the National Institutes of Health (Jaspreet Singh). National Institutes of Child Health and Human Development (NICHD) P50HD103538 (Ali Fatemi), Run for ALD and Brian's Hope Foundation (Ali Fatemi, Ann B. Moser, Bela Rui Turk).

## CONFLICT OF INTEREST

Potential competing interest for Ali Fatemi: Paid member of drug safety monitoring board – Bluebird Bio, Co‐inventor of Compositions and methods for treatment of peroxisomal disorders and leukodystrophies, Patent #US20170119899A1. Potential competing interest for Bela Rui Turk: Co‐inventor of Compositions and methods for treatment of peroxisomal disorders and leukodystrophies, Patent #US20170119899A1. Laila Marie Poisson, Christina Linnea Nemeth, Jordan Goodman, Richard Owen Jones, and Jordan Goodman have no relevant potential competing interests to report.

## ETHICS STATEMENT

The study was approved by the Johns Hopkins Medicine Institutional Review Boards (2005–2012 protocol 86‐03‐06‐01; 2013–2018 protocol NA_00045735) and Henry Ford Hospital IRB#12159.

## PATIENT CONSENT

Informed consent was given by the patient or his guardian prior to inclusion.

## Supporting information


**Figure S1.** Select molecules identified as differential between mild and moderate/severe AMN groups (ANOVA) are plotted with the estimated linear relationship (solid black line) and 95% confidence bands (gray) for (A) metabolites and (B) MiRNA. The range of observed molecular intensity for controls is denoted with green‐dashed lines.

**Figure S2.** Ordinary least squares regression of AMN patient age versus global score for clinical severity EDSS (*r* ^2^ = 0.25, *p* = 0.06).

**Figure S3.** KEGG analysis of mild versus moderate/severe AMN. (A) System impact and significance, highlighted systems above 0.4 impact. (B) Ranking of associated disease system.

**Table S1.** Baseline characteristics of patients and controls included in the study.

## Data Availability

De‐identified data will be made available upon reasonable request.
