# Supplementary figures and images for: MicroRNA and metabolomics signatures for adrenomyeloneuropathy disease severity
Source: JIMD Rep. 2022 Aug 22;63(6):593–603. doi: 10.1002/jmd2.12323 (PMC9626672; doi:10.1002/jmd2.12323)

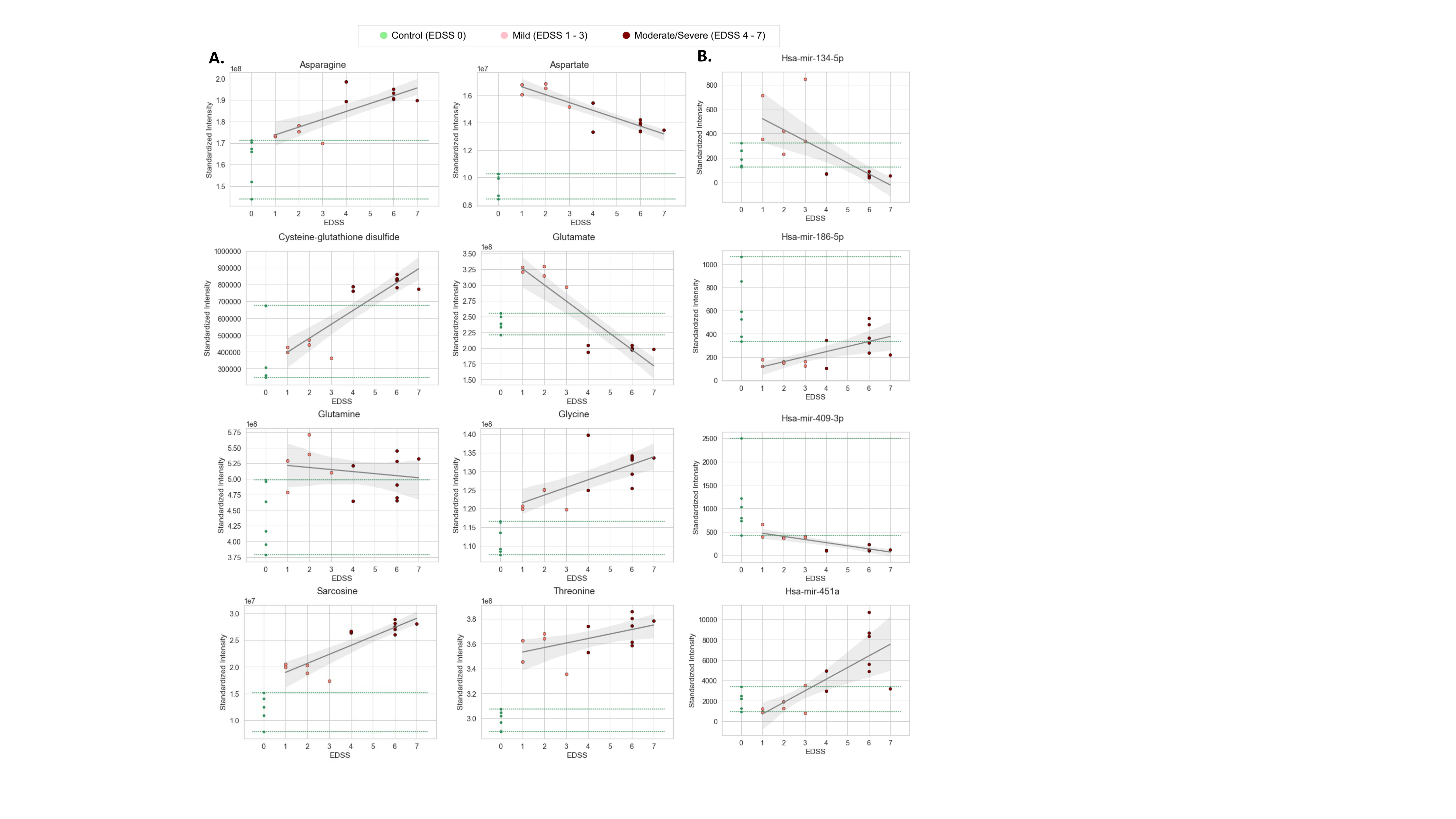

Supplement: Supplementary file 1 — Figure S1. Select molecules identified as differential between mild and moderate/severe AMN groups (ANOVA) are plotted with the estimated linear relationship (solid black line) and 95% confidence bands (gray) for (A) metabolites and (B) MiRNA. The range of observed molecular intensity for controls is denoted with green‐dashed lines. [file JMD2-63-593-s001.tif]

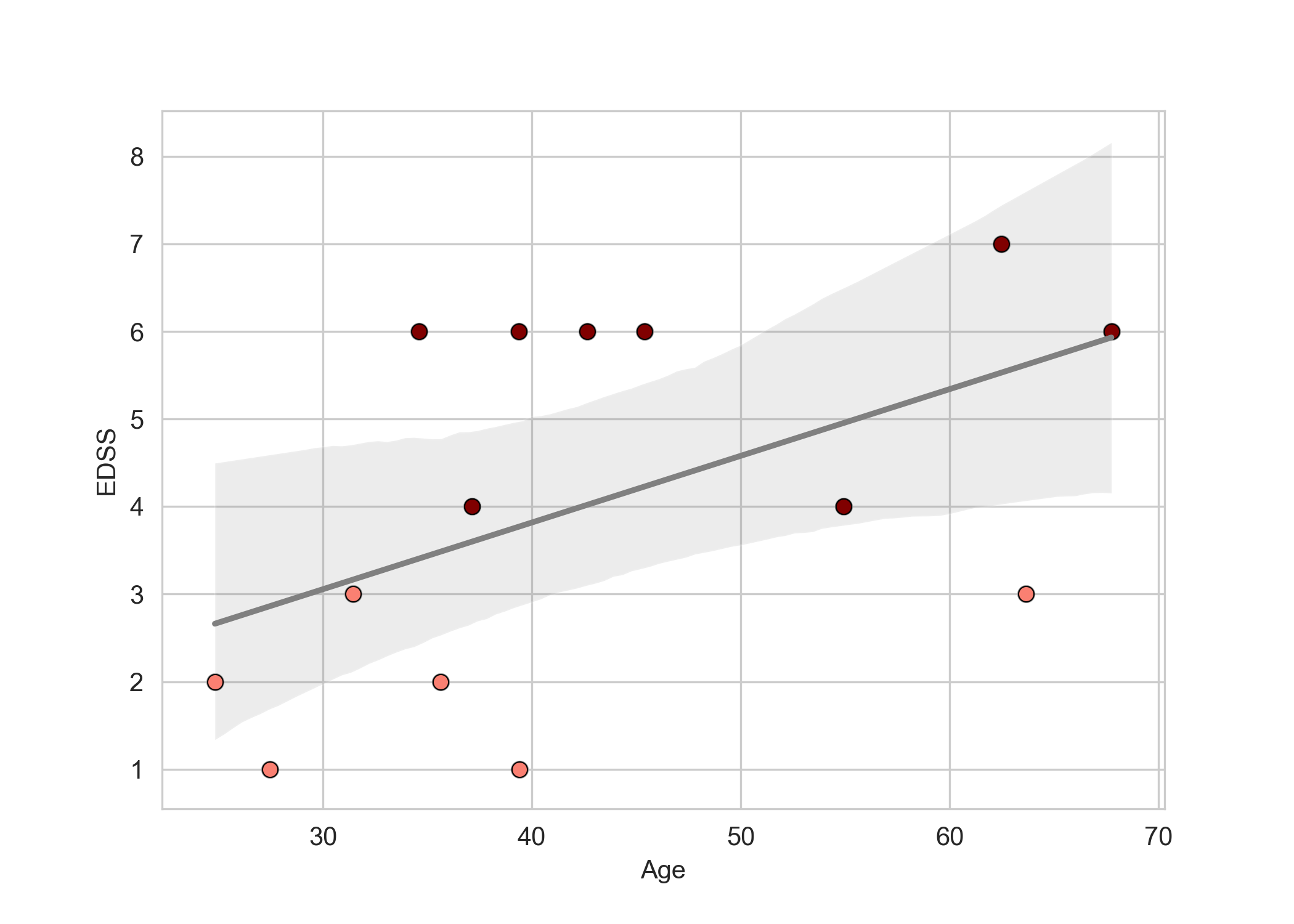

Supplement: Supplementary file 2 — Figure S2. Ordinary least squares regression of AMN patient age versus global score for clinical severity EDSS (r 2 = 0.25, p = 0.06). [file JMD2-63-593-s004.tif]

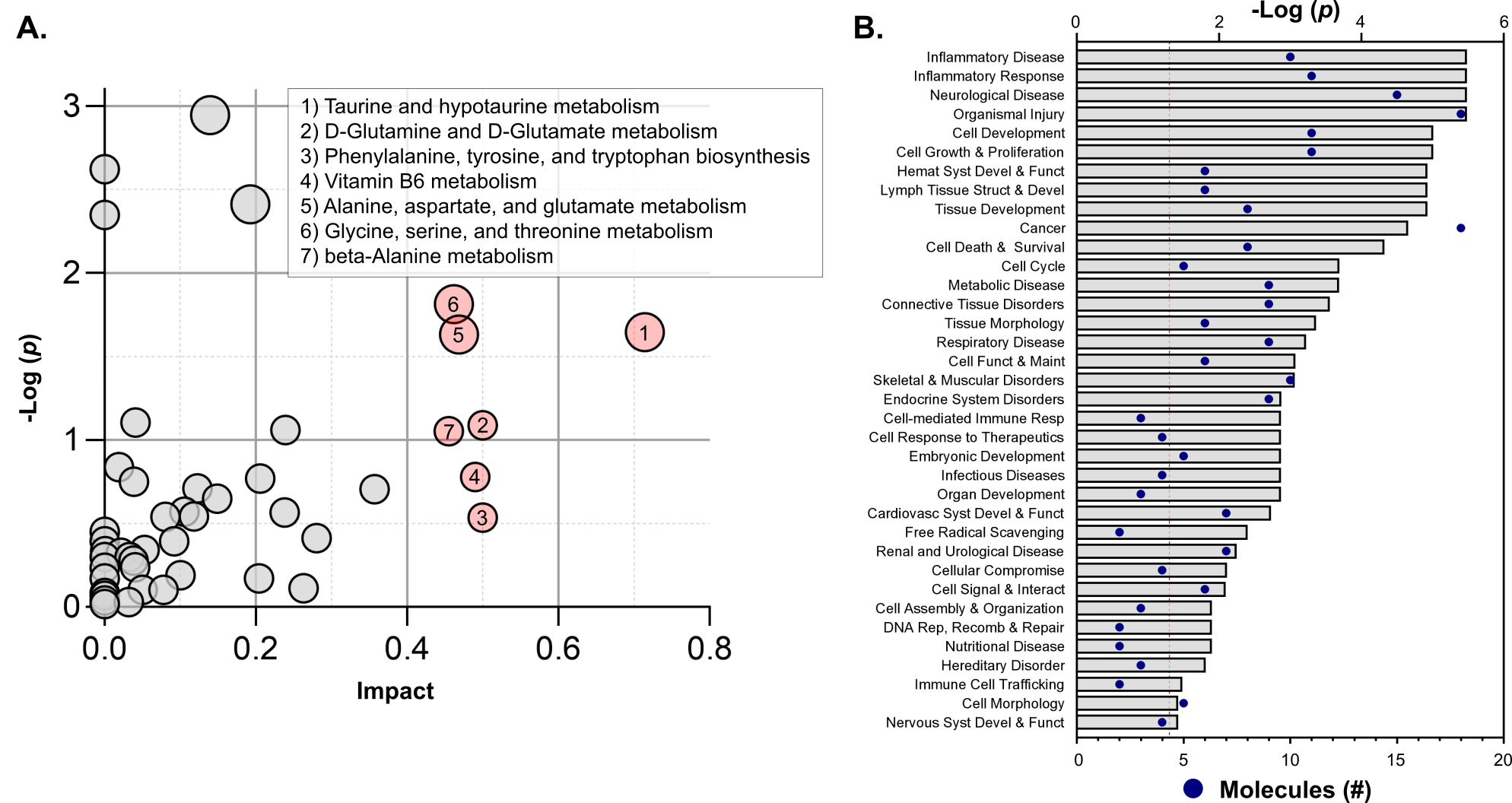

Supplement: Supplementary file 3 — Figure S3. KEGG analysis of mild versus moderate/severe AMN. (A) System impact and significance, highlighted systems above 0.4 impact. (B) Ranking of associated disease system. [file JMD2-63-593-s002.tif]
