# Supplementary material for: MicroRNA and metabolomics signatures for adrenomyeloneuropathy disease severity
Source: JIMD Rep. 2022 Aug 22;63(6):593–603. doi: 10.1002/jmd2.12323 (PMC9626672; doi:10.1002/jmd2.12323)
Supplement: Supplementary file 4 — Table S1. Baseline characteristics of patients and controls included in the study. [file JMD2-63-593-s003.docx]

**Supplemental Table 1**

| **Phenotype** | **Sample size** | **Age (Range, Stdev)** | **EDSS  (Range, Stdev)** |
| --- | --- | --- | --- |
| Control | 6 | 42.2 (24-73, 18.8) | 0 |
| Mild AMN | 6 | 37.1(27-63, 12.82) | 2 (1-3, 0.81) |
| Moderate & Severe AMN | 8 | 47(34-67, 11.47) | 6.2 (4-7, 0.99) |
